# Evaluation of caregiver-friendly workplace policy (CFWPs) interventions on the health of full-time caregiver employees (CEs): implementation and cost-benefit analysis

**DOI:** 10.1186/s12889-017-4722-9

**Published:** 2017-09-20

**Authors:** Allison M. Williams, Emile Tompa, Donna S. Lero, Janet Fast, Amin Yazdani, Isik U. Zeytinoglu

**Affiliations:** 1 School of Geography and Earth Sciences, McMaster University, General Science Building, 209, 1280 Main Street West, Hamilton, ON L8S 4K1 Canada; 2 Institute for Work and Health, University of Toronto, 481 University Avenue, Suite 800, Toronto, ON M5G 2E9 Canada; 3 Department of Family Relations and Applied Nutrition, Macdonald Institute, University of Guelph, 227A, 50 Macdonald Street, Guelph, ON N1G 1Y1 Canada; 4 Department of Human Ecology, University of Alberta, 2-06 Agriculture Forestry Centre, Edmonton, AB T6G 2P5 Canada; 5 Department of Kinesiology, University of Waterloo, 200 University Avenue West, Waterloo, ON N2L 3G1 Canada; 6 Conestoga College, 200 University Avenue West, Waterloo, ON N2L 3G1 Canada; 7 Department of Human Resources and Management DeGroote School of Business, McMaster University, 405, 1280 Main Street West, Hamilton, ON L8S 4M4 Canada

**Keywords:** Intervention research, Workplace accommodations., Mixed methods., Knowledge translation., Health.

## Abstract

**Background:**

Current Canadian evidence illustrating the health benefits and cost-effectiveness of caregiver-friendly workplace policies is needed if Canadian employers are to adopt and integrate caregiver-friendly workplace policies into their employment practices. The goal of this three-year, three study research project is to provide such evidence for the auto manufacturing and educational services sectors. The research questions being addressed are: What are the impacts for employers (economic) and workers (health) of caregiver-friendly workplace policy intervention(s) for full-time caregiver-employees? What are the impacts for employers, workers and society of the caregiver-friendly workplace policy intervention(s) in each participating workplace? What contextual factors impact the successful implementation of caregiver-friendly workplace policy intervention(s)?

**Methods:**

Using a pre-post-test comparative case study design, Study A will determine the effectiveness of newly implemented caregiver-friendly workplace policy intervention(s) across two workplaces to determine impacts on caregiver-employee health. A quasi-experimental pre-post design will allow the caregiver-friendly workplace policy intervention(s) to be tested with respect to potential impacts on health, and specifically on caregiver employee mental, psychosocial, and physical health. Framed within a comparative case study design, Study B will utilize cost-benefit and cost-effectiveness analysis approaches to evaluate the economic impacts of the caregiver-friendly workplace policy intervention(s) for each of the two participating workplaces. Framed within a comparative case study design, Study C will undertake an implementation analysis of the caregiver-friendly workplace policy intervention(s) in each participating workplace in order to determine: the degree of support for the intervention(s) (reflected in the workplace culture); how sex and gender are implicated; co-workers’ responses to the chosen intervention(s), and; other nuances at play. It is hypothesized that the benefits of the caregiver-friendly workplace policy intervention(s) will include improvements in caregiver-employees’ mental, psychosocial and physical health, as well as evidence of cost-benefit and cost-effectiveness for the employer.

**Discussion:**

The expected project results will provide the research evidence for extensive knowledge translation work, to be carried out in collaboration with our knowledge transition partners, to the employer/human resources and occupational health/safety target populations.

**Trial registration:**

ISRCTN16187974 Registered August 25, 2016.

**Electronic supplementary material:**

The online version of this article (10.1186/s12889-017-4722-9) contains supplementary material, which is available to authorized users.

## Background

In the past century, the most significant demographic transition in Canada that has revolutionized the labour force is the increase in female participation. This has been beneficial to the Canadian economy, but regrettably has had an impact on stress-related illness, such as cardiovascular disease; once a predominantly male pathology, it now accounts for at least 41% of all deaths of Canadian women in comparison to 37% for men [1]. Higher levels of stress for working women due to role strain negatively impacts their health and well-being [1]. Like paid labour, unpaid labour in the form of family care is also gendered, largely due to persistent gendered expectations that women assume domestic duties, compounded with gendered beliefs that women are inherently better at and more suited to caregiving than men. Moreover, women caregivers spend more hours per week involved in care tasks and typically are more likely to be engaged in care activities that must be done in person on a regular basis (e.g., personal care, food preparation) [2]. The majority of caregiver-employees (CEs) are women, and their participation in the workforce provides both social and financial support to their unpaid caregiving role, although the latter is linked with reduced mental and physical health [3]. Similarly, fulfilling the challenges of cultural and familial obligations while engaging in paid labour can lead to psychological stress, less time for self-care, and role conflict [4], as is seen in many cultures where women are expected to sacrifice their own needs for the needs of their husband, children, elderly parents, and in-laws.

The CE, faced with managing simultaneous paid work and unpaid care responsibilities, often encounters role strain (having to balance multiple work-family roles and responsibilities), forcing them to sacrifice one of their two roles and/or face negative health outcomes. These negative health outcomes which result from role strain can have a great impact on CEs’ health. CEs are defined as family members or friends who are providing care to an elder care recipient (often at home), while also engaging in paid employment. This new category of worker, created through numerous demographic changes and community health care provision realties of the twenty-first century, has brought attention to the need for support for family members and friends providing unpaid care. One strategy is through caregiver-friendly workplace policies (CFWPs). CFWPs, often termed family-friendly workplace policies, are defined as “deliberate organizational changes – in policies, practices, or the target culture – to reduce work-family conflict and/or support employees’ lives outside of work” [5]. Current Canadian evidence illustrating the health benefits and cost-effectiveness of CFWPs is needed if Canadian employers are to adopt and integrate CFWPs into their employment practices; otherwise they will continue to be reluctant to introduce CFWPs.

Since the 1990s, there has been an increased recognition of the need for financial and job security for caregivers [6–9], as well as relief from the role strain of what has been termed ‘the double day’ or the ‘second shift’. Workplace overload can lead to spill-over effects at home, and home stress due to caregiving can lead to negative effects at work, particularly for women who do not disengage as much after work compared to men [10]. With caregivers in Canada providing an estimated $26 billion of unpaid work, the Canadian health care system relies on informal unpaid care to support an aging population [11]. To address the problem of financial and job security, the Canadian government has implemented a number of programs for caregivers, such as the Compassionate Care Benefit (CCB) and caregiver and other tax credits. However, despite these attempts to provide informal unpaid caregivers with much-needed financial support and respite, policy-makers are coming to the realization that this is not enough. Rather, support needs to come from employers as well [7, 12–15]. For employers, there are several advantages to creating CFWPs. The potential costs of these policies can be offset by increased employee retention and less employee turnover, reduced absenteeism and presenteeism (i.e., engagement in work), and a positive company reputation, which can be beneficial both for business and for employee recruitment [2, 16–20]. In 2007, Canadian businesses lost over $1.3billion in productivity, as a result of informal unpaid caregivers missing full days of work, missing hours of work, or even quitting or losing their jobs [21]. Thus, from a business perspective, employers have much to gain financially from offering CFWPs, while simultaneously decreasing role strain, negative health outcomes and risk of occupational injury for CEs. This not only improves CEs’ job satisfaction but positively impacts CEs’ overall health [22–31]. Considering that informal unpaid caregivers play a significant, dual economic role in Canadian society, it is imperative to seek strategies that minimize or alleviate inequitable caregiver burden and the potential negative health outcomes which can arise for CEs [32].

Despite their potential benefits, many companies do not currently offer CFWPs [15]. The literature suggests a variety of reasons, including: the lack of initiative on the part of employers; the perceived challenges of implementing CFWPs; or organizational limitations, such as a small staff or a lack of human resources (HR) personnel [17, 33–35]. Studies have shown that employers with a relatively large staff, the presence of an HR department, and women in high-ranking senior positions are more likely to implement CFWPs because they have the knowledge, experience, and resources to do so [17, 31, 33, 34]. Workplaces that have a high use of technology are also more likely to accommodate CEs, as technology often lends greater work flexibility [36]. While different CFWPs have been used to varying degrees in almost every industry, the financial (banking, accounting, insurance) and public sectors currently appear to be the most innovative and advanced [17, 33, 34]. The manufacturing, transportation and construction sectors are the least developed, perhaps owing to the male-dominated workforce of many of these industries. Industries that are typically dominated by men may not view caregiving as an issue that their employees will encounter (given gendered assumptions about caregiving), and thus may not be aware of the value of implementing CFWPs [34].

Within the research literature, a variety of CFWPs have been identified as either being currently in use by selected employers across the globe, or proposed as potentially beneficial policies. From this review of literature, the most commonly identified CFWPs include: flexible work schedules; reduction of hours to part-time, and; unpaid care leaves (at time periods beyond the government-mandated timeframe) [34, 37]. Other CFWPs include: job-sharing; working from home (either part-time or full-time); transfer to a different branch that is closer to home; and the ability to work compressed work weeks [36]. Further, certain employers have also hired external companies or appointed HR staff to provide support services, such as: resource referral; information services; counseling; support groups; workshops; and seminars on caregiving issues [17, 34]. In exemplary cases, employers will offer extensive support in the form of: case management services; subsidized caregiving services; adult day care facilities; emergency short-term care; dependent care; flexible spending accounts; web-based support groups or seminars; and dependent-care car parks [34]. Clearly, such CFWPs require the re-allocation, if not re-investment, of resources by employers, whether HR, monetary, or other. Such CFWP initiatives begin with employers’ willingness to examine the research evidence that points to positive health outcomes for CEs.

Lack of workplace support for CEs can result in: CEs leaving the workforce, missed work days, early retirements, reduced productivity, and avoidable costs to employers. Employers need to step up to the challenge of managing a workforce which is, due to health care restructuring, increasingly expected to provide care to family members, often at home. Recognizing that all workplaces will be affected by these caregiving demands, and given population aging, the changing and dynamic nature of families (i.e. greater proportion of females in the workforce, smaller family sizes, increased mobility), together with the changing nature of caregiving (i.e. growing number of male caregivers, fewer publically-provided community services), it is incumbent upon Canadian workplaces to put in place CFWPs to appropriately accommodate CEs. Doing so is not only ethical but provides many advantages for the workplace, particularly given predicted skilled labour shortages. Uptake of CFWP intervention(s) by workplaces will likely depend on a number of factors such as funding availability, priorities in other domains, and precedence. Invariably, a key piece of information is the resource implications (both costs and consequences) of the chosen intervention(s). Knowledge about the resource implications of intervention(s) alternatives is critical. It provides invaluable information for making choices regarding investments of scarce resources, and is often a key input to the decision of whether to go forward with, expand, or downsize a particular intervention(s).

Given the context of the gendered nature of both paid employment and unpaid family care work, CFWPs exist at the intersection between sex/gender, work and health. As such, this project is framed within gender relations theory. Gender relations theory posits the need to better understand health-related behaviours in the context of interactions within and between women and men across numerous settings, including the personal, interpersonal and institutional levels [38]. As described by Bottorff et al., [38] a gender relations approach recognizes the central role of gender relations in health, including gender dynamics and the interactions thereof, in influencing both health opportunities and constraints. Connell’s gender relations theory is particularly suited to the phenomenon of working-caregiver strain, because it conceptualizes gender relations as being dynamic and performed daily via social interactions and practices [39]. In order to properly understand where and how CFWPs can be implemented, it is necessary to consider how both paid work and unpaid care are impacted by sex/gender. This can help ensure that CFWPs are able to reach the majority of CEs, while also best accommodating CFWPs to meet the needs of a growing number of male CEs. Moreover, CFWPs will likely differ depending on the presence or absence of sex/gender-dominance within a given industry. The results of this research will address a critical information gap, thereby providing evidence to employers about the importance of supporting CEs as a means of improving health, as well as reducing avoidable business costs. Framing two of the studies within gender relations theory will be coupled with an intersectionality approach to analysis [32], allowing for the examination of a number of axes of difference, such as sex/gender, employment status, ethnicity, geography, and income status, to be examined simultaneously.

## Methods

Three objectives will be addressed in three studies, Study A: Pre-Post Test of CFWP Intervention(s); Study B: Economic Evaluation of CFWP Intervention(s); and Study C: Implementation Analysis. These three studies share a number of commonalities, including using a comparative case study approach. The two participating employers represent different sectors of the workforce, making a strong argument for a comparative case study research design for all three studies, and particularly so for Studies A and C. The two participating employers represent the educational and auto manufacturing sectors in Canada. The features of a case study design benefit from complex and context-dependent research, such as the lived experience of CEs participating in the intervention(s). These design features include: comprehensive (longitudinal) and flexible data collection; data and investigator triangulation; comparability; and ability to select purposive examples for maximum diversity (i.e. in composition and variation). Written informed consent will be received for all participants recruited into this study. The qualitative data collected from each of the two of the studies (A and C) will be organized independently and stored via a computerized qualitative data management programme, NVivo10. All audiotaped qualitative data will be transcribed verbatim, imported into NVivo10, and thematically analyzed by the investigative team. The quantitative data collected via Study A and B will be analyzed separately using SPSS.

Each of the three studies will be conducted by a separate graduate student trainee, under the supervision of the Primary Investigator (PI) and, in the case of Study B, a Co-Applicant. Given that the studies will be implemented concurrently, the three graduate student trainees involved will have many opportunities for communication and exchange specific to each of their projects; this will allow them to learn from one another to assure timely success and productivity.

### Study a (objective 1): Pre-post test of CFWP intervention(s)

Using a pre-post test comparative case study design, Study A will determine the effectiveness of newly implemented CFWP intervention(s) across the two identified workplaces to determine impacts on CEs’ health. The intervention(s) will operate like a natural experiment, where a quasi-experimental pre-post design will allow the CFWP intervention(s) to be tested with respect to impact on CEs mental, psychosocial and physical health.

The intervention(s) will be chosen predominantly in consultation with the workplaces involved (i.e. with employers, HR departments, occupational health and safety representatives, and employees, etc.). This process is scheduled to take the first two months of the first year. Here, the research team will bring forward a summative report from an international synthesis of CFWPs, providing each employer partner team with a wide range of CFWPs from which to choose. The bi-weekly meetings, held throughout the initial two-month period of the grant (four meetings in total), will also determine which particular component of the workforce in each of the respective workplaces will be targeted for the research intervention(s), given the gendered nature of caregiving and the knowledge accrued specific to the age range where this is particularly salient (40–65 years).

Defined by the sample size calculation (See Additional file 1) [40], 37 CE participants in each workplace will be recruited into the study. This will provide a total sample of 74 participants across both workplaces. In addition to collecting socio-demographic data on participants, including sex and gender (via the BSRI), non-invasive health measures will be captured in both the pre- and post-implementation stages, including: self-reported health (SF12), self-rated burden (SRB), general self-efficacy (GSE), stress (RSS), mental health (CES-D), and psychosocial health (CRA). If sex and/or gender are not evenly represented in the participant sample, sex- and/or gender-specific CEs will be targeted. Within the 12 month intervention period, the time between the pre- and post-implementation stages will be reflected by the uptake/application of the intervention(s), both quantitatively (i.e. number of CEs using the intervention(s)) and qualitatively (i.e. intensity of use). Given the experience of other such intervention(s) studies, we hypothesize a minimum 25% increase in self-reported health (SF12). Given the size of each of the workplaces, we do not expect to have any difficulties recruiting 37 CEs participants per workplace. Even so, we will recruit as many CE participants as possible as a contingency plan. (see Additional file 1 for a range of sample size possibilities).

The quantitative data collected in Study A will be analyzed by way of descriptive statistics and ordered logit regression. SPSS Statistics software will be used (already purchased and in place at McMaster University). Summary statistics (including a differences between means test and contingency tables) will be produced to test for significant differences (*p* = 0.05) between pre and post-test data points. Tests will also be carried out to assess differences between CEs in each of the two workplaces concerned. A series of ordered logit regressions will be carried out to examine the association between levels of self-reported health – SF12 (the dependent variable) and the collected socio-economic variables (the independent variables). The regression models will assess differences in outcomes between CEs employed in various types of work (i.e. secretarial versus management versus assembly) in each of the two workplaces. The results of this analysis will inform the qualitative portion of the research.

A sub-sample of participants in each workplace will be invited to participate in a qualitative comparative case study (*n* = 20 CE participants; 10 in each workplace) to gather audio-taped, semi-structured in-depth interview data on the experience of having participated in the intervention(s), capturing the effectiveness of the intervention(s) outside of the quantitative variables examined in the pre-post test. Following the post-test data collection point, CE participants will be asked about specific ways in which the intervention(s) have impacted their experience of role strain, caregiver burden and overall health. The interviews will be transcribed verbatim. Guided by an intersectionality approach, this research will address meaningfully the ways in which social categories, such as sex/gender, and employment status intersect to impact CE health.

It is hypothesized that the benefits of the CFWP intervention(s) will include a number of health improvements for CEs, such as a decrease in role strain and improvements in CEs’ mental, psychosocial and physical health.

### Study B (objective 2): Economic evaluation of CFWP intervention(s)

Study B will focus on evaluating the resource implications of the CFWP intervention(s) undertaken in the auto manufacturing and educational services sectors in order to respond to the following question: What are the incremental costs and consequences of the intervention, relative to the status quo (the “do nothing” alternative), from organizational and societal perspectives? As an organizational perspective will only capture costs and consequences accruing to the employer/workplace, this research stresses the importance of a broader and societal level perspective in order to capture the full impact of the intervention to all stakeholders.

The types of economic evaluations to be undertaken are a cost benefit analysis (CBA) and a cost-effectiveness analysis (CEA). The CBA/CEA will analyze the costs and benefits and effects of each intervention(s) chosen for implementation in each of the respective workplaces compared to the status quo (i.e., the “do nothing” alternative). This is a common comparator used in workplace health evaluation when new intervention(s) are being evaluated [41–43]. The resources to be considered in the analysis will include the costs associated with making the intervention(s) available (people time, other resources costs, advertising expenditure, etc.), as well as those gained as a result of fewer sick days taken by caregivers, higher at-work productivity, lower turnover, continued labour force engagement, less use of health services, etc. The process that the CBA/CEA will follow is comprised of: selecting the intervention(s) and comparator time period; identifying stakeholders; measuring all known and quantifiable costs and consequences flowing from the intervention(s); predicting the outcome of cost and benefits/effects over a relevant time period; converting all costs and benefits into a common currency and effect into a common metric; applying a discount rate; calculating the net present value as well as other summary measures, such as the payback period and benefit-to-cost ratio, cost per unit of effect, executing a sensitivity analysis for key values; and reporting findings to the employers and workplace parties.

A before-after study design will be used, in which the before period in the same organization is used as the comparator (i.e., the “do nothing alternative”). CBA/CEA will be undertaken in which all resource implications of the intervention (both costs and consequences) are translated into monometer terms. For CEA, resource implication except the primary effect/outcome will be translation into monetary terms. A stop-and-drop approach will be used in which only those costs and consequences incurred during the measurement time period are considered, as well as a more comprehensive approach to intervention evaluation in which projections are made into the future about the resource implications of the intervention.

This study will attempt to capture as many of the resource implications (i.e. personnel costs, start-up costs, etc.) that follow from the chosen intervention(s), regardless of to whom they fall. Cost and consequences for each of the key stakeholders (i.e. CEs, employers, and society) will also be considered. Given that this study uses a CBA, there will inevitably be some resource implications of importance that are not adequately captured because they are intangible or not measured well in dollars; CEA allows us to keep at least the primary effect/outcome in natural units. This is particularly useful if the effect is better captured by a non-monetary metric, e.g., health. Stakeholders will play a key role in the identification and assessment of the valued resource implications and their relative importance in monetary and non-monetary terms.

In order to control for non-intervention(s) factors that may influence relevant outcomes in the before and after periods, regression modeling analysis (specifically, interrupted time series analysis) for key outcome variables will be undertaken, in which the unit of analysis will be the department per unit of time (often weekly or monthly time units depending on the size of the organization and data availability). Data for explanatory and outcome variables will be drawn from both primary data collection, as well as administrative data sources (i.e. sick days used, vacation days taken, etc.) made available by the employer partner organizations. Regression modeling allows us to control for contextual factors such as changes in the amount of hours worked by the unit, output changes, turnover, and external environment factors. The model will identify both the significance of the intervention(s) impact on the outcome of interest (e.g., sick days used, work/production output), as well as the magnitude of it impact. This information will be used to quantify the consequences of the intervention(s) in terms of changes to the outcome variable, which will then be translated into monetary terms, in the case of CBA, for use in the economic evaluation.

The expected outcomes will include information on the cost and consequences of the CFWP intervention(s) for different stakeholders. The resource implications of interventions are often a critical piece of information for key organizational decision makers, such as employers. Specifically, the impact of an intervention on the financial bottom line can influence whether to invest in, continue with, or expand a particular activity. Knowledge on the societal level economic impacts is also critical since it provides public sector decision makers with an understanding of the spillover effects of CFWPs to other stakeholders, and the merits of providing public support to encourage societally valued interventions.

### Study C (objective 3): Implementation analysis

Framed within a comparative case study design, Study C will undertake an implementation analysis of the CFWP intervention(s) in each participating workplace in order to determine: the degree of support for the intervention(s) (reflected in the workplace culture); how sex and gender are implicated; co-workers’ responses to the chosen intervention(s); and other nuances at play. An implementation analysis is a type of qualitative research study that investigates the determinants involved in the intervention(s)’ deployment and application; the effects of the intervention(s); and the influence of contextual factors on both the application and the effects of the intervention(s). This will translate into knowledge regarding the promotion of equity in the appropriate diffusion and availability of suitable CFWP innovation(s) for both the participating employers, the sectors they represent, and beyond.

Ethnography is appropriate for an implementation analysis as the researcher needs to be fully immersed in the ‘culture’ of the workplace. An ethnographic approach examines a small sample of participants in great depth over an extended period of time. This study will use maximum variation purposive sampling to recruit participants. During the proposed 12 months of fieldwork, data will be generated using participant observation and face-to-face interviews, in order to capture the richness of experiences while gaining insight into the dynamics of the social relations that exist within their historical, geographical, occupational, and cultural contexts. Participant observation will vary by week, but will average two days at each workplace. Here the researcher will observe and interact with the respective employees at break and lunch periods, as well as participate in all regular and special face-to-face meetings regarding the intervention(s), including those scheduled with the employer and research team. The audio-taped, semi-structured in-depth interviews will involve the intervention(s) participants (*n* = 5), as well as the associated human resource personnel (*n* = 3), managers (*n* = 2), administrators (n = 2), as well as co-workers (n = 3). In total, it is estimated that 15 face-to-face, audio-recorded in-depth semi-structured interviews will be conducted in each workplace (total sample will be 30). Each group of employees (intervention(s) participants, HR personnel, managers, administrators, co-workers) will be asked a unique set of questions regarding the implementation of the intervention(s), based on their location within the workplace. Following research ethics approval, these interviews will take place after the initial six month intervention(s) period (that is, half-way through the Study A intervention(s) period of 12 months). This will allow each group of employees to become familiar with the intervention(s) implementation process. The participant observation diaries and in-depth interviews (the latter which will be transcribed verbatim) will be stored and managed in NVivo10. Following a thematic analysis approach, the researchers will examine the textual data thoroughly, through multiple readings. During the readings, recurring themes will be documented in an iterative process.

It is hoped that this study will provide strategies on how to better implement the CFWP intervention(s) in both the workplaces concerned, as well as in other workplace settings. Guided by the thematic analysis approach to data interpretation, and with sex and gender at the centre of the analysis, this research will build on existing research on CFWPs intervention(s) and help in determining employment supports, such as CFWPs, that will assist in mitigating the negative impacts on CEs’ health and physical, psychosocial and mental well-being. Further, the results of the implementation analysis will allow other employers to learn what contributes to the success of CFWP intervention(s). The expected project results will provide the research evidence for extensive knowledge translation (KT) work, in collaboration with the identified KT partners, to the employer/human resources (HR)/occupational health and safety target population.

## Discussion

Given the strong recognition of the importance of the interface of policy, research and community, this project proposes to undertake a number of KT strategies beyond the traditional academic channels. With the assistance of a part-time Research Assistant specifically dedicated to KT, four specific groups of knowledge users will be targeted in order to disseminate the research evidence, so that it can be used to inform further uptake of CFWPs: (1) Canadian employers/HR personnel; (2) occupational health and safety organizations; (3) caregiver-employees; and (4) government and policy/decision makers. KT strategies are discussed in more detail below. KT goals include: integration of sex/gender sensitive CFWPs into workplaces characterized as employing CEs, and educating and advocating for CFWPs. Further, all research outcomes will be made available for dissemination via: (a) the PIs web-page [44]; (b) the web-based technology and network made available by The Canadian Center for Occupational Health and Safety (CCOHS), as well as; (c) hyperlinked on the appropriate web page of the two participating employers, as well as the union which represents employee members in both workplaces.

In addition to a final scientific and lay report, a minimum of five high impact academic conference presentations and three open-access peer-reviewed papers will be realized, targeting reputable occupational health and human resources venues across Canada. End-of-project KT will include a face-to-face KT Community Workshop on Caregiver-Employees, which will also be accessible virtually for those unable to attend in person. This KT Community Workshop will be a half-day meeting where the research results will be presented and strategically discussed, with respect to the desired outcomes specific to the KT and uptake of CFWPs. Invited attendees include: research team members, knowledge user/KT partners, study participants, and the general public. The workshop participants will receive a briefing paper of the results one week before attending the workshop. This will allow them to think through the results while answering pointed questions that will encourage discussion and uptake of the accrued knowledge around CEs and appropriate CFWPs. The outcomes of this workshop will be summarized in the final report to the funder, as well as disseminated via the web-based technology and network made available by all KT partners’ web pages.

## Additional file

Additional file 1: Calculation for a pre-post study design. This additional file outlines the formula used to calculate the sample size for this research. (PDF 66 kb)

## Abbreviations

BSRI: Bem Sex-Role Inventory
CBA: Cost benefit analysis
CCB: Compassionate Care benefit
CCOHS: Canadian Center for Occupational Health and Safety
CE: Caregiver-employee
CEA: Cost-effectiveness analysis
CES-D: Mental health scale
CFWP: Caregiver-friendly workplace policies
CRA: Psychosocial health scale
GSE: General self-efficacy scale
HR: Human resources
KT: Knowledge translation
PI: Principle investigator
RSS: Stress scale
SF12: Self-reported health scale
SRB: Self-rated burden scale
