# Supplementary material for: Evaluation of caregiver-friendly workplace policy (CFWPs) interventions on the health of full-time caregiver employees (CEs): implementation and cost-benefit analysis
Source: BMC Public Health. 2017 Sep 20;17:728. doi: 10.1186/s12889-017-4722-9 (PMC5607563; doi:10.1186/s12889-017-4722-9)
Supplement: Additional file 1: — Calculation for a pre-post study design. This additional file outlines the formula used to calculate the sample size for this research. (PDF 66 kb) [file 12889_2017_4722_MOESM1_ESM.pdf]

Additional File 1

Calculation for a Pre-Post Study Design

The formula used to calculate the required sample size for a pre-post study design is:

$$n=D[(Z_\alpha+Z_\beta)^2*(p_1(1-p_1)+p_2(1-p_2))/(p_1-p_{(2)})^2]$$

Where,

n = required minimum sample size

D = design effect

P1 = the estimated level of an indicator measured as a proportion at the time of the first survey

P2 = the expected level of the indicator either at some future date

Z_α = the level of statistical significance that the observed change of size would not have occurred by chance

Z_β= the statistical power

Based on the published research by Fast et al. (2013), the proportion of CEs who had 'Excellent/very good' self-access health (SAH) was 60%. We will use this as the proportion at the time of the first interview. Using standard parameters of: 95% level of significance, and; 80% power, and employing a one-tailed test (since we want to test whether the implementation of caregiver-friendly workplace polices (CFWP) intervention(s) improves SAH. Suppose an increase of 25% in the proportion of having 'Excellent/very good' SAH is to be measured at the second interview, after the CFWP intervention(s) implemented. In this case, p1=0.6 and p2=0.8; Z_α=1.96 and Z_β=0.84; D=2 because a cluster sampling design is chosen given the selected

employers. The use of a standard value of D=2.0 should adequately compensate for the use of cluster sampling.

n = 2 [(1.645+ 0.840)^2 * ((.6)(.4) + (.85)(.15)) / (.85 - .6)^2]

=2[6.175*0.3675/0.625] = 72.6

Given this, 73 participants are needed to evaluate the CFWP intervention(s). Given that we will use a cluster sampling strategy, an estimated 37 participants are needed per employer partner organization (this provides a total of 74 participants).

Table 1: Sample sizes required for selected combinations of P1 and detected changes

[95% significance level and 80% power, D=2(cluster sampling)]

	Change/difference to be detected (P2 - P1)					
P1	0.05	0.1	0.15	0.2	0.25	0.3
0.10	1074	309	152	93	63	45
0.15	1420	389	185	110	73	51
0.20	1717	457	213	124	81	56
0.25	1964	513	235	134	86	60
0.30	2161	556	251	142	90	62
0.35	2309	587	262	147	92	62
0.40	2408	605	268	148	92	62
0.45	2458	611	268	147	90	60
0.50	2458	605	262	142	86	56
0.55	2408	587	251	134	81	51
0.60	2309	556	235	124	73	45
0.65	2161	513	213	110	63	38
0.70	1964	457	185	93	51	29
0.75	1717	389	152	73	37	19
0.80	1420	309	114	49	21	7
0.85	1074	216	70	23	3	
0.90	679	111	21			

Note: Sample sizes shown assume a design effect (D) of 2.0. Suppose it was desired to show a 10% increase of a health outcome, from 20% to 30%, initial level of the indicator, or P1, would then be 0.20 (see left hand column), and the sample size would be 457.
